# Composition and Properties of Protective Coatings Made of Biologically-Derived Polyester Reactive Binder

**DOI:** 10.3390/polym13111700

**Published:** 2021-05-22

**Authors:** Szymon Kugler, Ewa Wierzbicka, Paula Ossowicz-Rupniewska, Jakub Łopiński

**Affiliations:** 1 Department of Chemical Organic Technology and Polymeric Materials, Faculty of Chemical Technology and Engineering, West Pomeranian University of Technology in Szczecin, Piastów 42, 71-065 Szczecin, Poland; paula.ossowicz@zut.edu.pl (P.O.-R.); jakub.lopinski@zut.edu.pl (J.Ł.); 2 Łukasiewicz—Industrial Chemistry Institute, Rydygiera 8, 01-793 Warsaw, Poland; ewa.wierzbicka@ichp.pl

**Keywords:** rosin, polyester, bio-diol, halloysite, powder paint

## Abstract

Biologically derived polymers are a very attractive subject for investigation, due to the strict pro-ecological requirements imposed by developed countries, including zero-waste and zero-carbon policies as well as volatile organic compound (VOC) limits. Synthesis of biologically-derived polyesters from natural rosin and bio-diols, showing softening temperatures suitable for application in VOC-free paints and varnishes, was performed to create a desired, future commercial product, that meet the aforementioned requirements regarding VOC and elimination of petroleum-based raw materials. Prepared polymers were used in the formulation of coating materials whose properties: cross-linking behavior, glass transition temperature, thermal stability, storage modulus, hardness, cupping resistance, adhesion, chemical resistance, gloss, haze, color, and anti-corrosive behavior in the salt chamber were investigated and discussed. As a result, coatings with prepared bio-polyesters contained over 80 wt.% of natural resources and showed competitive/better properties than petroleum-based references. They can be applied in the prototyping of “green” powder paints for the protection of steel substrates from corrosion and aggressive solvents.

## 1. Introduction

Annual production of polymers grows by ca. 3% per year exceeding 438,000,000 tons in 2017 [1]. They constitute a colossal and complex amount of chemical products, which can be classified in various ways, one of which is division into three huge groups of products, having one-dimensional (fibers), two-dimensional (coatings, films), and three-dimensional (castings) morphologies. Among the mentioned materials, the coatings generate an extraordinary amount of problems sourced in the presence of volatile organic compounds (VOC) in the coating compositions (i.e., paints and varnishes) and petrochemical origin of polymer binders, which terribly contrasts with modern waste-free and zero-emission policies, currently being implemented in all developed countries [2].

One of the best ideas to avoid VOC emissions from coating compositions is a powder composition. Powder coatings belong to the group of one-component coating materials in the form of fine-grained (<100 µm) powder. They are intended mainly for the protective coating of metal substrates. When powder paint is applied to a substrate, using special application methods such as electrostatic spraying, the coated detail is heated at a high temperature in order to melt the paint and, usually, perform the cross-linking process [3]. The world production of powder coatings will soon exceed three million tons per year, and the market value will reach USD 12.5 billion by 2022 [4]. Such good economic indicators for this market are due to the fulfillment of strict requirements regarding the emission of volatile organic compounds (which is a problem in the case of liquid paints) and the fact that powder coatings are characterized by a significantly better performance than liquids in terms of excellence of finish, coating efficiency and economy of production [3]. Due to low cost and high performance, epoxy-polyester powder coating systems constitute the vast majority of the growing, but highly competitive powder coatings market [3,4].

The major problem to solve in the field of powder paints is the market need of low-price and high-performance products causing the minimal carbon footprint under the most actual carbon neutrality policy. Expectations related to the implementation of the carbon-neutral economy in the US, China, and EU by 2050 pile up the formal obstacles and increasing costs for paint producers, forcing them to seek biobased alternatives [5,6,7,8,9].

Such a biobased alternative is rosin: a cheap, abundant, and non-toxic raw material, which can be easily converted into many valuable chemicals, including solid epoxy resins and hardeners [10]. They are based on rigid diterpene skeletons that give them a significantly better performance than other biobased counterparts in terms of mechanical, thermal and barrier and functional features [5,7,9,11,12,13,14,15]. The mentioned rosin derivatives were characterized by a considerably growing interest in recent years both in scientific and patent literature. That interest was focused on novel, rosin-based resins and polymers obtained from such already known rosin intermediates as maleopimaric acid, dehydroabietyl chloride, acrylpimaric acid and dehydroabietylamine, as well. Their application included not only curing agents and resins, but also bio-active polymers, elastomers, coatings, adhesives, surfactants, sorbents and many other materials. Nevertheless, epoxy-cured two-component materials constituted the most well-described group of rosin derivatives in recent years. The problem is that although several rosin-derived components [16,17,18,19,20,21,22,23,24,25,26,27,28] curable in epoxy-ring-opening reactions have been prepared so far, they do not meet the requirements for use in two-rosin-component powder paints, i.e.,

- the flexibility of chains in molecules enabling non-brittle curing of coatings;
- and softening temperatures in a range of ca. 100–140 °C, which should be lower than curing temperatures of powder coatings (usually 140–220 °C).

In this work, innovative rosin-biodiol polyesters with tailored melting temperatures have been synthesized in order to use them as polyester resins with free carboxyl groups in novel powder paints that can be cured with solid epoxy resins, including rosin glycidyl ester product. Moreover, the mechanical, thermal, and functional properties of coatings in comparison with petroleum-based reference samples have been evaluated. Furthermore, the influence of the addition of a natural filler—halloysite—was also investigated. Such a selection of components allowed to prepare compositions with natural resources contents up to 83 wt.%.

## 2. Materials and Methods

### 2.1. Materials

The following raw materials were used without further purification:

A. Halloysite (Dunino, Poland) color RAL 9001, specific surface ca. 53 m^2^·g^−1^, average lumen inner diameter 11 nm, average particle diameter 93 nm. B. Rosin (Alfa Aesar, Haverhill, MA, USA) containing ≥75 wt.% of abietic acid and used without further purification. In fact, a mixture of abietic acid and other natural resin acids (such as pimaric acid, sandaracopimaric acid, levopimaric acid, dehydroabietic acid, neoabietic acid, and others—identified by GC-MS after conversion to methyl esters) was used for the research. C. 1,4-butanediol (BASF, Ludwigshafen, Germany) 99%. D. 1,10-decanediol (Acros Organic, Geel, Belgium) 99%. E. Maleic anhydride (Acros Organic, Geel, Belgium) 99% pastilles. F. Dibutyltin oxide (Fascat 4201, Brenntag, Essen, Germany). G. 3-ethanolamine (Merck, Darmstadt, Germany) 99%. H. t-butylammonium bromide (Merck, Germany) ≥99%. I. 8-hydroxyquinoline (Merck, Darmstadt, Germany) ≥99%. J. Epichlorohydrin (Solvay, Brussels, Belgium) ≥99%, bio-based. K. Diethyl ether (Chempur, Piekary Śląskie, Poland) ≥99,9%. L. n-hexane (Chempur, Piekary Śląskie, Poland) ≥99,9%. M. Sodium hydroxide (Chempur, Piekary Śląskie, Poland) >98,8%. N. Acetone (Chempur, Piekary Śląskie, Poland) ≥99,9%. O. Xylene mixture (Chempur, Piekary Śląskie, Poland) ≥99,9%. P. Commercial bisphenol-A-based epoxy resin (CER) showing T_m_ = (96–100) °C and epoxy equivalent weight EEW = (867–930) g/eq, (Ciech, Nowa Sarzyna, Poland). Q. Commercial curing agent P-108 (CH) dedicated to solid epoxy resins (Hexion, Stuttgart, Germany).

### 2.2. Synthesis of Maleopimaric Acid and its Triglycidyl Ester

**Figure 1:**
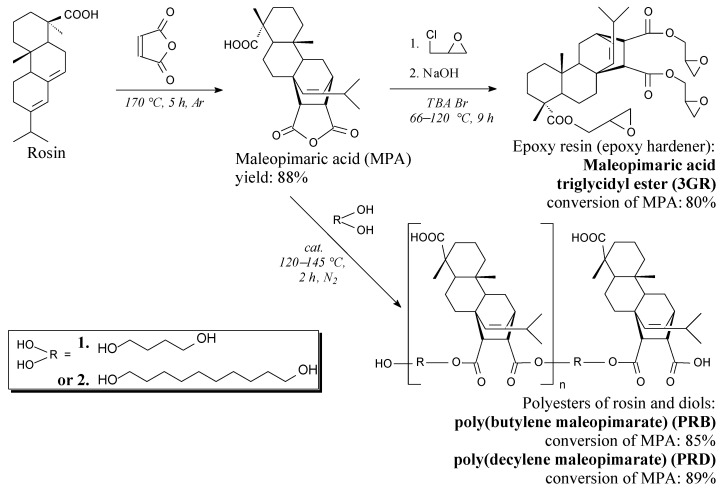
Preparation routes of rosin-derived chemicals: polyester resins PRB, PRD, and epoxy hardener 3GR.

General synthesis methods of maleopimaric acid (MPA) and maleopimaric acid triglycidyl ester (3GR), and its spectroscopic and some physiochemical properties were presented in Supplementary Data. Syntheses of MPA [16] and 3GR [20] had been also reported previously. Reaction schemes were shown in Figure 1.

### 2.3. Synthesis of Rosin-Biodiol Polyesters

**Figure 2:**
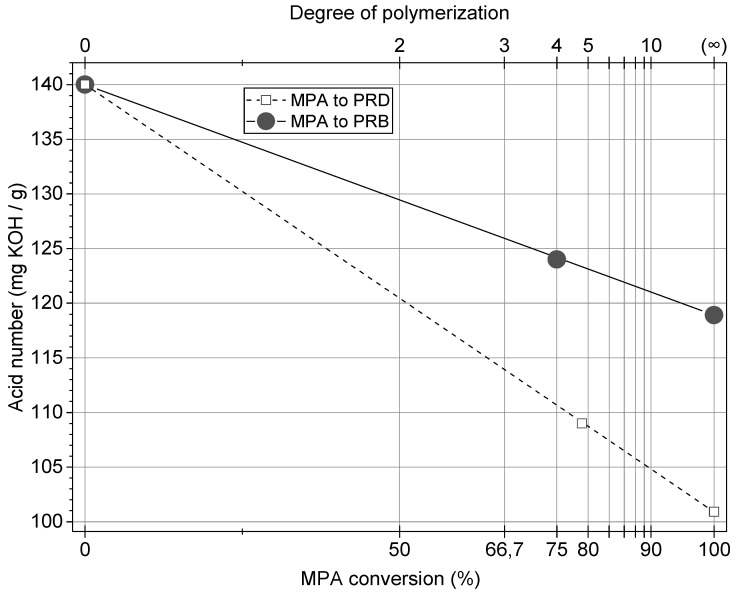
Calculation of MPA conversion and degree of polymerization of PRD and PRB polyesters based on the acid number.

The synthesis of rosin-biodiol polyesters was carried out in a 100 cm^3^ glass reactor equipped with a stainless steel stirrer, distillation column, and inert gas inlet. A mixture of maleopimaric acid (1 moL), diol (1 moL), and a catalyst (0,2 wt.% per amount of reacting mixture) was placed in the reactor and heated under a nitrogen atmosphere. Two types of a catalyst—3-ethanolamine or dibutyltin oxide—were applied. The reaction was carried out at a temperature of 120–145 °C in an organic solvent, xylene. The reaction mixture was heated for 2–3 h. After that xylene was distilled. The obtained polyester resins were characterized by the determination of the acid number and softening point temperature; the results are presented in Table 1. The theoretical values of polymerization degree were calculated basing on acid number regression in the function of MPA conversion, which was shown in Figure 2.

### 2.4. Preparation of Natural Origin Anti-Corrosive Filler

Anti-corrosive filler was prepared in a two-stage process, widely described in our previous work [29]. The process was briefly described in Supplementary Data.

### 2.5. Preparation of Natural-Resources-Rich Powder Coatings

Each powder coating composition was obtained as follows. Components: resin, hardener, and filler were ground into fine powders using a knife mill. The proportion of resin to hardener was stoichiometric. Next, they are mechanically pre-mixed in stoichiometric proportions (optionally in presence of 2,5 wt.% of filler) before extrusion via twin-screw (16 mm, l/d = 40) co-rotating extruder Prism 16 (ThermoFisher, Waltham, MA, USA) at 90 °C, 60 rpm. After cooling in ambient conditions, the extrudate was ground by knife milling (1000 rpm) and classified using a sieve shaker. A fraction of 45–65 µm was loaded to Optiflex-2 corona-charging gun (Gema, St. Gallen, Switzerland), applied uniformly onto steel substrates, and then cured in an oven at 180 °C for 15 min. The measured coatings thickness was 100 ± 15 µm. The acronyms and composition of prepared coatings are summarized in Table 2.

### 2.6. Evaluation Methods of the Components, Coating Compositions, and Coatings

The acid number of polyesters was determined according to EN-ISO 2114:2005. The method was based on the KOH titration.

Softening point temperature was determined using the ring-ball method according to ASTM D36-66.

The Brunauer-Emmett-Teller specific surface areas of the halloysite materials were measured by low-temperature nitrogen adsorption using a TriStar II 3020 V1.03 (Micromeritics Company, Norcross, GA, USA).

Complex viscosity of coating compositions was tested using DHR-1 rheometer equipped with Environmental Test Chamber and 25 mm plate-plate measuring system (TA Instruments, New Castle, DE, USA), gap 1 mm, 1 Hz at 0,1∏ rad oscillation, heating from 90 to 200 °C (heating rate 10 °C min^−1^) followed by the reaction under isothermal conditions. Additionally, after cooling down to 25 °C, storage modulus values of cured compositions were saved.

Differential Scanning Calorimetry (DSC) was performed on a Q-100 device (TA Instruments, New Castle, DE, USA). 9–13 mg samples were heated from 25 °C to 240 °C with a heating rate of 10 °C min^−1^, standard aluminum pans were used under nitrogen atmosphere 25 cm^3^∙min^−1^, the reference was an empty pan.

Thermogravimetric analysis (TGA). The analysis of cured coatings was carried out on a Q-5000 device (TA Instruments, New Castle, DE, USA) in a platinum crucible. Samples between 5–10 mg were heated from 25 °C to 1000 °C with a heating rate of 10 °C min^−1^, under air atmosphere, flow rate: air—25 cm^3^∙min^−1^, nitrogen (as protective gas)—10 cm^3^∙min^−1^.

Ultraviolet-Visible Spectroscopy (UV-Vis) was done using UV9000s device (Biosens, Warsaw, Poland) for determination of coatings color of samples on 5 cm × 5 cm substrates. The color was recorded using the device firmware and converted to standard RAL charts.

Gloss and haze of coatings at 20 °C were determined using IQ20/60/85 device (Rhopoint Instruments, St. Leonards, UK), in the automated measurement of randomly chosen points of coatings, in compliance with the ISO 2813 standard, 5 measurements.

The hardness of coatings was tested using AWS-5 König pendulum (Dozafil, Wrocław, Poland), 20 °C, 50% of relative humidity, 5 measurements.

Adhesion of coatings to steel was checked in a cross-cut test, and assessed in a 6-grade scale from 0 (the best) to 5 (the worst) adhesion, according to EN ISO 2409 standard, 3 measurements.

Cupping resistance (ISO 1520) of coatings on steel was determined using Model 200 cupping tester with a hard ball pressed into the left (non-coated) side of the substrate (Erichsen, Hemer, Germany), 3 measurements.

Chemical resistance to methyl ethyl ketone (MEK) was investigated in rubbing test, according to EN 13523-11 standard, 3 measurements.

Performance in the salt chamber was carried out according to PN-EN ISO 9227:2007 in CorrosionBox 400 (Co.Fo.Me.Gra., Milano, Italy) using an aqueous NaCl solution (concentration of 50 ± 5 g/L) sprayed with compressed oil-free air (100 kPa). The backside and edges of steel QD-46 Q-panels (dimensions: 102 mm × 152 mm) with x-cut paints (according to EN ISO 17872:2007) were protected with a special pressure adhesive tape (Tesa, Charlotte, NC, USA) and mounted at an angle of 20° vertically. The temperature in a spray cabinet was maintained at 35 °C during the test period of 1000 h.

## 3. Results and Discussion

### 3.1. Properties of Rosin-Derived Polyester Resins

Two prepared polyesters of rosin and biodiol: poly (butylene maleopimarate)—PRB and poly (decylene maleopimarate)—PRD were prepared for the first time, according to the best of our knowledge. These polymers joined the group of “green” polyesters based on such raw materials as saccharides, polyhydroxyalkanoates, lactic acid, and others [13,30,31,32,33,34]. The reaction of polycondensation was carried out until acid numbers corresponding to the MPA conversion of 75–80% were achieved (Figure 2). Such values of MPA conversion were empirically determined to obtain resins showing softening temperatures adequate for, as mentioned in the Introduction, powder coating applications (Table 1): conversion values higher than 80% resulted in softening temperatures of resins higher than 140 °C, making them unsuitable for powder paint formulation. The achieved conversion values resulted in softening temperatures of 100–120 °C, which is optimal for the discussed application. It is noteworthy that the polyesters were not separated from unreacted monomers (MPA and bio-diols) but were used as solid resins in the formulation of innovative powder coatings with a high content of natural resources.

### 3.2. Thermal Properties of Coating Materials

**Figure 3:**
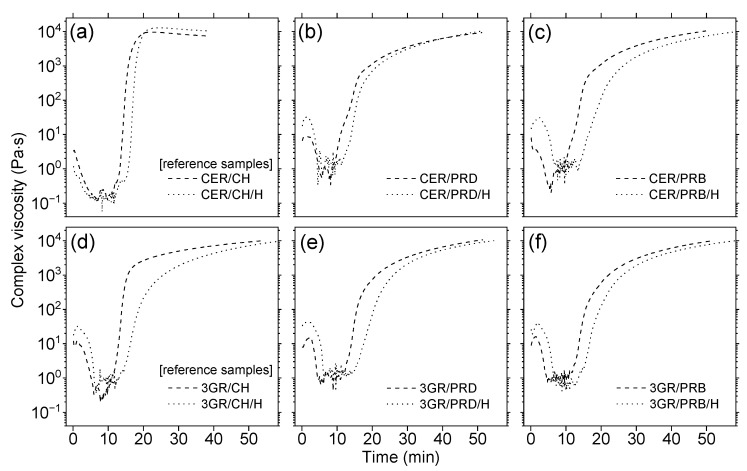
Rheological curves of the reference sample (**a**,**d**) and prepared coating composition (**b**,**c**,**e**,**f**) curing processes involving heating from 90 to 200 °C (heating rate 10 °C/min) followed by the reaction under isothermal conditions (200 °C). Symbols are explained in Table 2.

The investigation of thermal properties of prepared polymer coating materials included viscosity measurements of coating compositions, glass transition temperature (T_g_), and thermal stability of coatings. The results of complex viscosity changes in the function of time were shown in Figure 3. It should be mentioned here that the temperature dependence of time was simple: samples were heated up from 90 to 200 °C (for 11 min) followed by the reaction under isothermal conditions. The viscosity values of all the samples initially decreased below 1 Pa·s before rapid growth to values of ca. 10^4^ Pa·s. The time for reach-up maximum viscosity values was shorter for reference compositions from petroleum-based resources (Figure 3a) than for samples containing rosin derivatives (Figure 3b–f), especially PRB and PRD polyesters (Figure 3b,c,e,f). This can be explained by the lower mobility of large polyester macromolecules that contain rigid abietane structures (as can be seen in Figure 1) in comparison with simpler petroleum-based components. All samples modified with a natural filler (dash curves) showed a longer time to reach the 10^4^ Pa·s threshold than their unmodified counterparts (solid curves). It can be concluded that the use of natural components extended curing times due to the characteristic chemical structure of organic components, as well as the presence of inert particles of the filler, which were obstacles in polymerization and cross-linking processes. Nevertheless, all coating compositions were correctly crosslinked in less than 60 min, which is an acceptable value for potential customers. There is no available data on rheological measurements of the cross-linking of rosin-based compositions reported by others, whereas the influence of inert filler on retardation of the curing process is commonly known in the art, and is described in the scientific literature [35].

**Figure 4:**
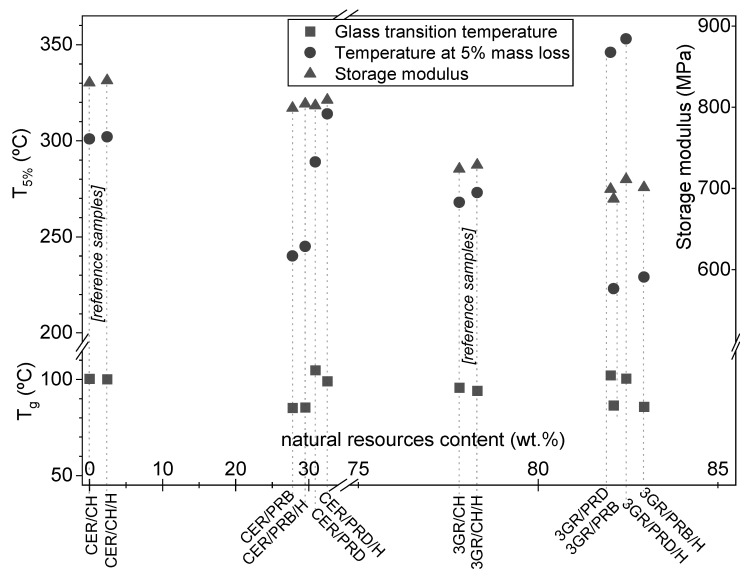
Content of natural resources in prepared coatings and reference samples and their values of glass transition temperature (T_g_) and temperature at 5% mass loss (T_5%_). Symbols are explained in Table 2.

The thermal stability of obtained samples was defined as the temperature at 5% mass loss (T_5%_). Its values were given in Figure 4 and were ordered according to the increasing content of natural resources in the coating. It should be elucidated here that the coatings prepared from rosin-based polyesters contained from 27 to 83 wt.% of natural resources. This means that they reached a very satisfactory natural component content compared to other studies [16,17,18,19,20,21,22,23,24,25,26,27,28]. It should be noted that the maximum content of natural resources (83 wt.%) resulted from the presence of maleic anhydride built in the structure of polyesters and 3GR hardener (Figure 1). Taking into consideration T_5%_ values of the polyester-based coatings, in relation to petroleum-based references, the discussed parameter had not deteriorated nor even improved for most PRD binders, while for samples containing PRB polyester, it was noticeably reduced. The highest T_5%_ value was reached for 3GR/PRD/H (349 °C), which meant ca. 50 °C improvement in comparison with the 300 °C value of the reference CER/CH/H sample (Figure 4). In contrast, samples based on PRB polyester exhibited significantly lower T_5%_ values in the range of 210–250 °C. The lower thermal stability of PRB-based coatings in comparison with promising PRD-based materials can result from a lower polymerization degree of poly (butylene maleopimarate) as compared to poly (decylene maleopimarate), which was shown in Figure 1. Furthermore, the addition of halloysite increased T_5%_ values of all samples (as can be seen in Figure 3), which is a known in the art, and is an appreciable phenomenon based on heat transfer fundamentals. It is worth noting in this results discussion that results published by others also suggest that the higher the molar mass, the better the thermal stability of rosin-based materials [18,26]. It may be preliminarily concluded that a higher molar mass of rosin-based dianhydride can improve the thermal stability of samples.

On the other hand, T_g_ values of polyester-based coatings did not differ so significantly as the aforementioned T_5%_ values. As can be seen in Figure 4, coatings with natural resourced polyesters exhibited T_g_ values in the relatively narrow range of 85–105 °C, which were closed to bisphenol-A-based samples (ca. 100 °C). Lesser differentiation can result from the fact that rosin-based components were projected and prepared for powder coating application, so their reactivity (Figure 3) and phase transition behaviors (T_g_, Figure 4) were similar to the reference samples. It is noteworthy that samples with PRB polyester showed noticeably lower T_g_ (ca. 88 °C), whereas the presence of PRD polyester increased T_g_ values of samples over 100 °C. It suggested that PRB was less effective as a polyester resin, in contrast to PRD, which should be as effective as its referenced commercial counterparts. For the record, the influence of halloysite on the T_g_ thermal parameter was rather irrelevant.

### 3.3. Mechanical Properties of Coatings

**Figure 5:**
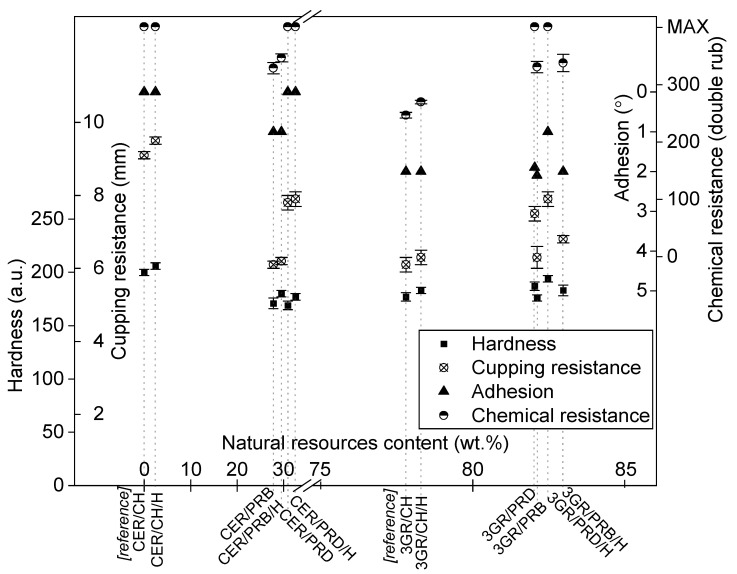
Hardness, cupping resistance, adhesion, and chemical resistance of prepared coatings and reference samples. Symbols are explained in Table 2.

Mechanical performance tests of prepared coatings included storage modulus, hardness and cupping resistance, which are particularly important in the protection of steel substrates. The results were shown in Figure 5, except storage modulus, which was presented in Figure 4. As can be seen, the storage modulus of prepared coatings was decreased with the growing content of natural resources from 830 MPa to ca. 600 MPa. Nevertheless, the presence of halloysite in each coating slightly increased the parameter values. A slightly lower modulus of elastic properties of rosin-based polyester materials was also observed by others [18,20]. Furthermore, polyester-based coatings were characterized by high pendulum hardness values in the range of 160–190 a.u. (compared to 230 a.u. that is the hardness of borosilicate glass surface); however, irrelevantly higher values of hardness were noted for petroleum-based commercial references. Slightly lower hardness values were recorded for samples with PRB polyester compared to PRD-based materials, while the addition of halloysite slightly increased the coatings hardness.

The reference ISO 1520 cupping resistance values [36] were in the range of 9,1–9,5 mm (samples CER/CH and CER/CH/H). All rosin polyester samples showed lower values of the mentioned parameter (between 6 and 8 mm). Moreover, the presence of halloysite in coatings noticeably improved their cupping behavior. Furthermore, taking into consideration the polymer in the coating structure, samples with PRD polyester showed better cupping resistance than materials with PRB.

Summarizing the presented results, the PRB-based samples showed a noticeably lower mechanical performance than materials with PRD polyester. Importantly, samples with polyesters exhibited mechanical properties that can be safely accepted on the powder coatings market. The presence of the natural filler was beneficial to all the coating systems, improving their hardness and cupping resistance too. Among the prepared rosin-based samples, the most advantageous mechanical features were noted for the 3GR/PRD/H sample, containing 82,5 wt.% of natural resources.

### 3.4. Functional Properties of Coatings

**Figure 6:**
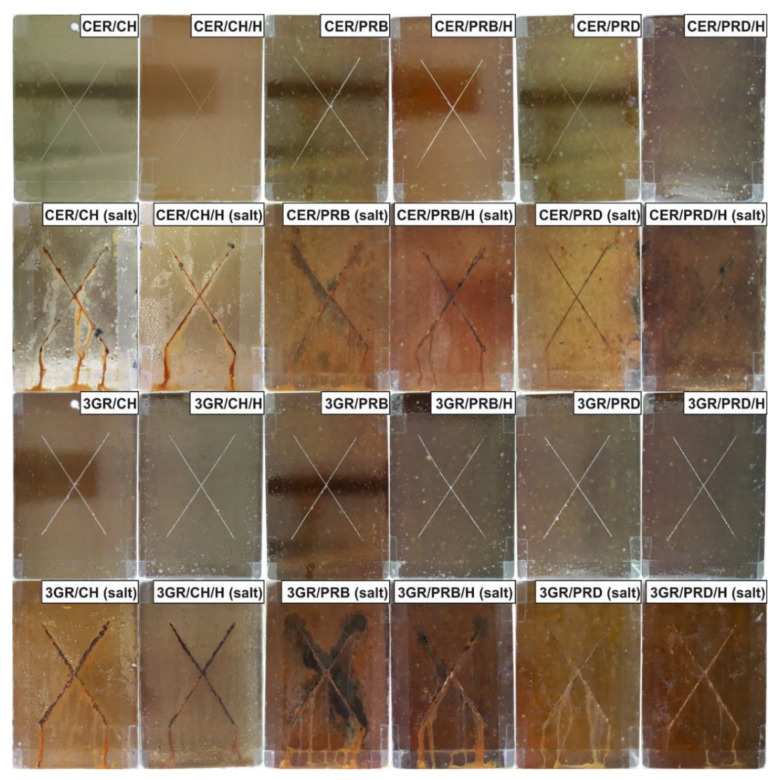
Prepared coatings and reference samples before and after 1000 h of salt spray test.

From the point of view of a powder coatings end-user, the most relevant functional properties are focused on protection and outlook. The most important protection-oriented functional properties—adhesion, chemical resistance, and behavior in a corrosive atmosphere—were chosen for this investigation. The results of ISO 2409 adhesion [37] were given in Figure 5. It was easy to find that, in general, this parameter decreased with the growing natural resources content. Nevertheless, some of the prepared coatings exhibited satisfactory adhesion of 0–1°; this included all CER/polyester samples and also 3GR/PRD/H. The halloysite did not visibly influence the discussed parameter, except for sample 3GR/PRD/H, which showed 1°, compared to 2° of 3GR/PRD. Advantageously, all the prepared coatings exhibited high results in EN13523 chemical resistance tests [38] whose results were set in Figure 5. Moreover, all samples with PRD polyester showed the best possible result in the mentioned test, achieving a maximum barrier performance against methyl-ethyl ketone (400 double rubs). The outstanding chemical resistance of several samples was accompanied by a good anti-corrosive performance, examined in a 1000 h salt spray test [39]. The results were presented in Figure 6. Petroleum-based reference samples CER/CH and CER/CH/H showed reference anti-corrosive performance: rusty X-scratch with medium trickles and some black corrosion centers. 3GR/CH and 3GR/CH/H reference samples were characterized by slightly worse anti-corrosive behavior, probably because a significant amount of corrosion products in X-scratch had been caused by insufficient adhesion (Figure 5). Among the samples with rosin-based polyesters, CER/PRD and CER/PRD/H exhibited very good performance with minimal corrosion located only in X-scratch, a few trickles, and some small black corrosion centers. On the other hand, the 3GR/PRD sample showed anticorrosive behavior resulting in numerous trickles; however, its version with halloysite (3GR/PRD/H) exhibited significantly fewer trickles. Anticorrosive performance of four samples with PRB polyester was noticeably worse than PRD-based samples, causing delamination and underneath corrosion of steel substrates. It is noticeable that the natural filler improved the anti-corrosive performance of all the samples. Therefore, the presence of a PRD polyester, as well as modified halloysite filler, proved to be beneficial for the protective properties of prepared coatings. It should be mentioned here that the choice of salt chamber test to investigate anti-corrosive features of coatings is preferred by commercial customers more than indirect methods, e.g., electrochemical impedance spectroscopy (EIS). Nevertheless, EIS is appreciated in basic research, so the prepared coatings should be examined via EIS as part of our future contribution.

The outlook-oriented features are particularly relevant for topcoat (decorative) applications, while their necessity for primers is less important. This group of functional properties of coatings included gloss, haze, and color as well. The results were presented in Table 3. Colors of unfilled samples, matching the RAL color chart, were described as shades of yellow, gold, grey, and brown, while coatings with modified halloysite showed grey and brown colors. It is worth noting, that the presence of natural halloysite contributed to the darkening of coatings color. The results of gloss and haze of prepared materials [40] showed, that the presence of natural components (rosin derivatives and/or halloysite) decreased their gloss and increased haze. Beneficially, all results of color and gloss were within the discretionary acceptability of the potential end-user.

Summarizing all the functional properties of prepared materials, three samples with bio-based polyester: CER/PRD, CER/PRD/H, and 3GR/PRD/H showed the most acceptable for end users compilation of good hardness, acceptable adhesion and cupping resistance, significant protection from corrosion, and solvent, as well as acceptable visual properties, that made them applicable as primers or also topcoats in an environment that are not exposed to UV radiation. Among them, 3GR/PRD/H was characterized by high natural resources content (82,5 wt.%) that should be an important factor in the development of described materials.

## 4. Conclusions

Two new rosin/biodiol polyesters: poly (1,4-butylene maleopimarate) and poly (1,10-decylene maleopimarate) were synthesized and applied in thermosetting protective coatings on steel substrates. Natural resources content in prepared materials was between 27 and 83 wt.%. Three coatings with PRD polyester (CER/PRD, CER/PRD/H, and 3GR/PRD/H) showed functional, mechanical and thermal properties competitive with the petroleum-based references. Moreover, 3GR/PRD/H sample was characterized by high natural resources content (82,5 wt.%) which proved that naturally sourced coating materials can replace their petrochemical-origin counterparts. Other advantages of this coating in comparison with reference materials were 40 °C better thermal stability, not deteriorated glass transition temperature (ca. 100 °C), high hardness, comparable/slightly better anti-corrosive performance in the industrial salt chamber, as well as very high chemical resistance. On the other hand, it showed longer cross-linking time (over 45 min), lower gloss, storage modulus and cupping resistance, and also higher haze but, fortunately, these 5 parameters were within the discretionary acceptability of potential end-users, especially taking into account its resistance to aggressive media and elevated temperature. Moreover, modified halloysite improved thermal stability, hardness, cupping resistance and anti-corrosive performance of prepared coatings. The mentioned advantages of use poly (1,10-decylene maleopimarate) as a resin in polyester/epoxy coating compositions allow us to claim, that this polymer can be a promising replacement of petroleum-based components in the formulation of powder coating binders. The next stage of development of these materials should be a composition of new paints based on 3GR/PRD/H binder with anti-corrosive pigments.

## Figures and Tables

**Table 1:** Characteristics of rosin derivatives used in the formulation of coating compositions.

Sample Symbol	Used Bio-Diol	Catalyst	Reaction Temperature (°C)	Softening Temperature (°C)	Acid Number (mg KOH/g)
PRB	butanediol	dibutyltin oxide	140 ± 5	100	190
PRD	decanediol	3-ethanolamine	125 ± 5	120	170

**Table 2:** Symbols and composition of prepared coatings and reference samples.

Sample Symbol	Weight Ratio of Components in Coating Composition (wt. Part)					
	Commercial Epoxy Resin (CER)	Rosin-Based Epoxy Resin (3GR)	Commercial Hardener (CH)	BD/Rosin Polyester (PRB)	DD/Rosin Polyester (PRD)	Modified Halloysite (H)
CER/CH ^a^	97.8	-	2.2	-	-	-
CER/CH/H ^a^	97.8	-	2.2	-	-	2.5
CER/PRB	65.7	-	-	34.3	-	-
CER/PRB/H	65.7	-	-	34.3	-	2.5
CER/PRD	61.9	-	-	-	38.1	-
CER/PRD/H	61.9	-	-	-	38.1	2.5
3GR/CH ^a^	-	93.7	6.3	-	-	-
3GR/CH/H ^a^	-	93.7	6.3	-	-	2.5
3GR/PRB	-	38.8	-	61.2	-	-
3GR/PRB/H	-	38.8	-	61.2	-	2.5
3GR/PRD	-	35	-	-	65	-
3GR/PRD/H	-	35	-	-	65	2.5

^a^—reference sample.

**Table 3:** Visual properties of prepared coatings and reference samples.

Sample Symbol	Gloss (G.U.)	Haze (a.u.)	Color (RAL)
CER/CH	70 ± 3	7 ± 1	7034 (yellow grey)
CER/CH/H	62 ± 4	10 ± 1	8000 (green brown)
CER/PRB	70 ± 4	6 ± 1	7008 (khaki grey)
CER/PRB/H	65 ± 2	8 ± 1	8008 (olive brown)
CER/PRD	63 ± 3	9 ± 1	1020 (olive yellow)
CER/PRD/H	50 ± 3	15 ± 1	7006 (beige grey)
3GR/CH	61 ± 2	9 ± 1	1036 (pearl gold)
3GR/CH/H	55 ± 3	14 ± 1	7002 (olive grey)
3GR/PRB	68 ± 1	10 ± 1	8024 (beige brown)
3GR/PRB/H	60 ± 5	16 ± 1	7013 (brown grey)
3GR/PRD	52 ± 3	14 ± 1	8025 (pale brown)
3GR/PRD/H	51 ± 2	16 ± 1	7006 (beige grey)

## Data Availability

Not applicable.

## Supplementary Materials

The following are available online at https://www.mdpi.com/article/10.3390/polym13111700/s1, Synthesis of maleopimaric acid (MPA); Synthesis of rosin-derived epoxy hardener (3GR).
