# Supplementary material for: Composition and Properties of Protective Coatings Made of Biologically-Derived Polyester Reactive Binder
Source: Polymers (Basel). 2021 May 22;13(11):1700. doi: 10.3390/polym13111700 (PMC8196985; doi:10.3390/polym13111700)
Supplement: Supplementary file 1 [file polymers-13-01700-s001.zip › polymers-1225301-supplementary.pdf]

Supplementary Data

Composition and properties of protective coatings made of biologically-derived polyester reactive binder

Szymon Kugler[1,*], Ewa Wierzbicka[2], Paula Ossowicz-Rupniewska[1] and Jakub Łopiński[1]

[1] West Pomeranian University of Technology in Szczecin, Faculty of Chemical Technology and Engineering, Department of Chemical Organic Technology and Polymeric Materials, Piastów Ave. 42, 71-065 Szczecin, Poland; paula.ossowicz@zut.edu.pl (P.O.-R.); jakub.lopinski@zut.edu.pl (J.Ł.)
[2] Łukasiewicz – Industrial Chemistry Institute, Rydygiera 8, 01-793 Warsaw, Poland; ewa.wierzbicka@ichp.pl
* Correspondence: skugler@zut.edu.pl

Synthesis of maleopimaric acid (MPA)

MPA was synthesized according to the previously described method [1]. For this purpose, 150 g of rosin and maleic anhydride in a molar ratio of 1.2:1 were introduced into a 500 cm^3 flask. Reactions were carried out at 170 °C for 5 h under argon. After this time, the reaction mixture was cooled to room temperature. The crude product was crystallized from a 1:2 diethyl ether/n-hexane solvent mixture. The resulting precipitate was filtered off under reduced pressure and then dried at 60 °C under reduced pressure (10 mbar) for 3 hours to obtain 109,31 g of light-beige, solid product (88% yield with respect to maleic anhydride). The reaction scheme of rosin conversion into MPA was presented in Figure 1.

Synthesis of rosin-derived epoxy hardener (3GR)

Maleopimaric acid triglycidyl ester (3GR) was synthesized in accordance with [2]. In a 1000 mL Duran glass reactor with mechanical stirring 200 g (0.5 mol) of MPA, 372 g (4 mol) of epichlorohydrin and 5 g of tetrabutylammonium bromide was heated to 120 °C and mixed for 5 h under nitrogen. Next, the mixture was cooled down to 66 °C followed by careful addition of 40 g (1 mol) of sodium hydroxide in 40 g of distilled water. The stirring was continued for 4 h under nitrogen at 70 °C. After that, the mixture was filtrated, washed 1:2 with hexane (3 times) and evaporated under vacuum at 60 °C to obtain 112 g of solid yellowish 3GR product having m.p. 70-73 °C and determined EEW

299-362 g·equiv^{-1}).The reaction scheme of MPA conversion into epoxy resin was presented in Figure 1.

Preparation of natural origin anti-corrosive filler

Anti-corrosive filler was prepared in a two-stage process widely described in our previous work [3]. In the first stage, 100 g of natural halloysite was operated in a demineralized water-loaded ultrasonic bath (35 kHz) at room temperature for 24 h. In the second stage, it was ultrasonically mixed with 8-hydroxyquinoline in demineralized water (35 wt.% suspensions) and stirred using a mechanical agitator in the ultrasonic field. The quantity of modifying compounds implemented in the process was taken through numerous preliminary experiments. Next, the solvent was evaporated, to transform the dry mass into a fine powder using a ball mill. The resulting filler parameters: specific surface 17 $m^2 \cdot g^{-1}$, average lumen inner diameter 21 nm, average particle diameter 250 nm.

References:

1. Liu, X.; Xin, W.; Zhang, J. Rosin-Based Acid Anhydrides as Alternatives to Petrochemical Curing Agents. *Green Chem.* **2009**, *11*, 1018–1025, doi:10.1039/B903955D.
2. Liu, X.Q.; Huang, W.; Jiang, Y.H.; Zhu, J.; Zhang, C.Z. Preparation of a Bio-Based Epoxy with Comparable Properties to Those of Petroleum-Based Counterparts. *Express Polym. Lett.* **2012**, *6*, 293–298, doi:10.3144/expresspolymlett.2012.32.
3. Wierzbicka, E. Modifier of Epoxy Coating Compositions Properties. Preparation and Characterization. *MATERIAL ENGINEERING* **2020**, *1*, 14–18, doi:10.15199/28.2020.5.2.
